# Decoding the vital segments in human ATP-dependent RNA helicase

**DOI:** 10.6026/97320630016160

**Published:** 2020-02-29

**Authors:** Vandana Kamjula, Ananya Kanneganti, Rohan Metla, Kusuma Nidamanuri, Sudarshan Idupulapati, Ashish Runthala

**Affiliations:** 1 Department of Biotechnology, Koneru Lakshmaiah Education Foundation, Vaddeswaram, AP, India

**Keywords:** RNA helicase, innate immunity, motif, MODELLER, flexibility

## Abstract

An analysis of the ATP-dependent RNA helicase using known functionally close analogs helps disclose the structural and functional information of the enzyme. The enzyme plays several interlinked biological functions and there is an urgent need to interpret its key active-site residues to infer function and establish role. The human protein q96c10.1 is annotated using tools such as interpro, go and cdd. The physicochemical properties are estimated using the tool protparam. We describe the enzyme protein model developed using modeller to identify active site residues. We used consurf to estimate the structural conservation and is evolutionary relationship is inferred using known close sequence homologs. The active site is predicted using castp and its topological flexibility is estimated through cabs-flex. The protein is annotated as a hydrolase using available data and ddx58 is found as its top-ranked interacting protein partner. We show that about 124 residues are found to be highly conserved among 259 homologs, clustered in 7 clades with the active-site showing low sequence conservation. It is further shown that only 9 loci among the 42 active-site residues are conserved with limited structural fluctuation from the wild type structure. Thus, we document various useful information linked to function, sequence similarity and phylogeny of the enzyme for annotation as potential helicase as designated by uniprot. Data shows limited degree of conserved sequence segments with topological flexibility unlike in other subfamily members of the protein.

## Background

RNA helicase is ubiquitously present in viruses, bacteria, archaea and eukaryotes, and is the largest cluster of enzymes linked with RNA metabolism [1]. Being a highly conserved enzyme, it plays a phenomenal role in the unwinding of the RNA duplexes [2] and requires the hydrolysis of nucleoside triphosphates [3]. The DEAH-box protein (DHX) family members are usually located in the nucleus region. The laboratory protein of genetics and physiology2 (LGP2) is a member of the DEAD-box protein family and belongs to the ATP-dependent RNA helicase family [4,5], known to be involved in various steps of RNA metabolism [6] with several pleiotropic functions [7]. The catalytic core of these proteins encodes 12 highly conserved motifs [8]. LGP2 is a key regulator of interferon-induced with helicase-C domain1 (IFIH1)/ melanoma differentiation associated protein5 (MDA5) and DExD/H-Box helicase58 (DDX58)/retinoic acid-inducible gene (RIG-I)-mediated antiviral [9, 10]. When the antiviral pathway gets perturbed, RIG-I usually initiates a cascade of deregulated events, which further causes the immunological disorders [11].It shows a significant response against several viruses including newcastle disease, rhabdovirus, sendai, lassa, orthomyxoviruses (influenza), ebola and flaviviruses (hepatitis). While it acts both against the single or double-stranded RNA, MDA-5 is active against the long double-stranded RNA and recognizes picornaviruses and vaccinia viruses. Both these proteins are shown to have an active response against dengue, West Nile and Japanese encephalitis viruses [12]. Thus, helicases play key roles in regulating the innate immune responses [13]. Active research is going on RNA helicase, and enormous articles have been published to date (September 14th, 2019) [14,15,16, 17,18,19,20,21, 22]. The two databases national center for biotechnology information proteins (NCBI) and universal protein resource knowledgebase (UNIProtKB) orderly contain 1335 and 422 sequences in contrast to 154 structures listed in the protein data bank (PDB). The ever-increasing sequence-structure gap for this protein makes its sequence, structure, conservation or phylogeny analysis quite elusive for the evolutionarily distinct human sequence variants. For its key role behind the regulation and control of gene regulation and RNA metabolism, there are growing implications for DHX subfamily in human diseases and their treatment [23,24]. It is of interest to report an analysis of the ATP-dependent RNA helicase using known functionally close analogs to help disclose the structural and functional information of the enzyme.

## Materials and Methods:

**Figure 1:**
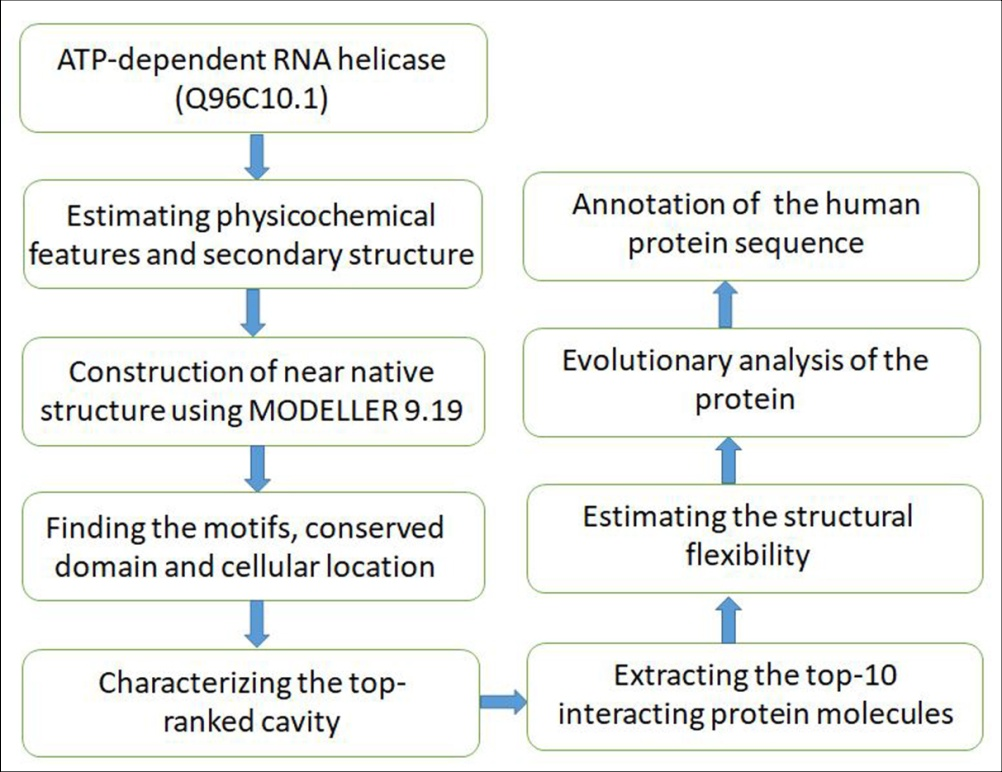
Flowchart showing the robust annotation algorithm for the human protein sequence. To ascertain the predictions, the methodology deploys the key sequence, structural and evolutionary measures.

For functionally characterizing the un-annotated human protein sequence, the following strategy is developed, as depicted through a flowchart in (Figure 1).

### Sequence retrieval:

The amino acid sequence of ATP-dependent RNA helicase (Q96C10.1) is retrieved from the UniProtKB/SwissProt database.

### Prediction of physicochemical properties:

Several features, viz. residue composition, molecular weight, theoretical PI, instability index, extinction coefficient, atomic composition, aliphatic index, and grand average of hydropathicity (GRAVY) score are essential to define the physicochemical properties and to estimate the structural features of a protein sequence. The parameters are estimated through the Expasy-Protparam tool (https://web.expasy.org/protparam/) [25].

### Secondary data prediction:

PSIPRED algorithm is deployed to predict the three-state secondary structure (Helix, strand, and coil). It provides credible information corresponding to α-helices, beta-sheets, coils, transmembrane helices, signal peptides, membrane interactions, re-entrant helix, and putative domain boundaries [26].

### Molecular modelling:

To construct a near-native structure of the RNA helicase sequence, HHPred [27] is used to screen the top-ranked functionally similar protein structure(s) (templates) from the PDB database by extending the sequence profile on the basis of 5 iterative rounds [28,29]. The template 5F9FE, sharing the highest sequence similarity of 53%, is selected and the protein model is built using MODELLER9.19 [30].The unaligned 1-residue N-terminal and 12-residue C-terminal (667-678) segments are truncated to curate the alignment file and to construct the 2-666 residue model structure. As the predicted decoy is found to encode several atomic clashes, it is energetically relaxed/refined through3Drefine [31],and the best model is selected on the basis of qualitative model energy analysis (QMEAN) and ERRAT scores. The model is assessed through the discrete optimized potential energy (DOPE) and GA341 scores of MODELLER. By using the QMEAN server, the predicted top-ranked model is assessed through the Molprobity score on the basis of rotamer outliers and the atomic clash score. Ramachandran map is subsequently plotted through the PROCHECK server to assess the topological accuracy of the predicted structure on the basis of phi and psi angles.

### Functional scrutiny:

The sequence is fed to InterPro server to retrieve the information regarding the superfamily, domains, repeats and gene ontology [32].Conserved domain database (CDD) is subsequently screened to affirm the credibility of the screened domains for purging the spurious hits/superfamilies and selecting the credible ones [33]. To estimate the interaction of the selected protein with the closely- related sequences, the STRING database is used [34]. For a robustly accurate analysis, its algorithm deploys several parameters including gene fusion, gene neighborhood, gene co-occurrence, text mining, and co-expression to estimate a confidence score. The score ranges between 0 and 1 and, for all the considered features, it is expected to remarkably score the closely interacting protein pairs.To localize the three most-conserved motifs, the MEME suite is used [35]. On the basis of gapless local alignment of multiple sequences (GLAM2) protocol, it even covers the gapped motifs [36]. The algorithm helps to identify DNA and protein sequence motifs. The default motif length range of 6-50 is used for the analysis. PROFUNC (http://www.ebi.ac.uk/thornton-srv/databases/Pro Func/) is further used to estimate the biochemical functions through the sequence homology against the PDB database [37]. To reliably affirm the intracellular/cytosolic locus of the human helicase protein, the hidden markov models-dependent server (TMHMM2.0 www.cbs.dtu.dk/services/TMHMM) is used [38]. Peptide cutter, a web-based tool, (https://web.expasy.org/peptide _ cutter/) is subsequently used to predict the location of probable cleavage sites of chemicals/proteolytic enzymes.

### Conservation and flexibility analysis:

To reliably affirm the sequence conservation profile of the sequence, UniProtKB/SwissPROT database is screened through HMMER for the selected protein [39]. With a very strict E-value inclusion cutoff of 0.00001, the sequence profile is expanded through five iterative rounds. From a total of 728 ATP-dependent RNA helicases, 259 sequences are selected. As the sequence length of experimentally solved protein structures is found to be within 600-800, the sequence length filter (580-820 residues) is conservatively used along with the removal of bifunctional proteins.Sequences are retrieved using Batch-Entrez and aligned by ClustalW module of HHpred. Consurf is subsequently used to track the degree of conservation across the chain [40]. Deploying the constructed sequence profile, the conservation scores are statistically estimated with the Bayesian probability across the chain on a scale of 1-9. To define the functional conservation across the chain, it takes input from sequence alignment and draws phylogeny connections among the sequences to plot it over the deployed/predicted reference structure through color gradations. Surface topography is further analyzed by computed atlas of surface topography of proteins (CASTp) to locate the active-site within the modelled protein structure [41]. It locates pockets, internal cavities, and the cross channels along with their surface area and volume, and reveals the functionally important sites within a protein structure. To study flexibility across the active-site and derive the root mean square fluctuation (RMSF) fluctuations across the cavity, the CABS-flex2.0 server is used [42]. It estimates flexibility/rigidity of the secondary structures and key residues of the constructed model. For localizing these flexible sites in correlation with the topology of the predicted model, Polyview-2D (http://polyview.cchmc.org/) server is used [43].

### Phylogeny analysis:

To draw a credible evolutionary analysis, Gblocks is used for eliminating the evolutionary divergent regions and poorly aligned segments from the constructed alignment. It removes the ambiguous regions and takes into consideration only the conserved regions to construct a phylogenetic tree. The resultant output is fed to a phylogeny server (http://phylogeny.lirmm.fr/phylo_cgi/ index.cgi) to construct an evolutionary tree [44,45]. Using the minimum value of SH-like statistically assesses the evolutionary relationship of the sequence dataset and Chi-2 based tests. The evolutionary distances are further computed using the Jonathan Taylor Thomas (JTT) matrix method.

## Results:

### Physicochemical properties:

The physicochemical properties of the ATP-dependent RNA helicase are estimated through ProtParam. For the 678-residue sequence, the molecular weight is estimated to be 76.6KDa. The sequence encodes 75 negatively and 73 positively charged residues, and it indicates that the protein is somewhat negatively charged. Theoretical pI is estimated to be 6.98 and it exhibits a slightly acidic nature. The extinction coefficient value and in-vitro half-life of the protein are respectively estimated to be 66,350 and 30 hours. The molecular formula is shown to be C_3365_H_5391_N_983_O_994_S_34c_and it shows the GRAVY score of -0.294.

### Secondary structure prediction:

**Figure 2:**
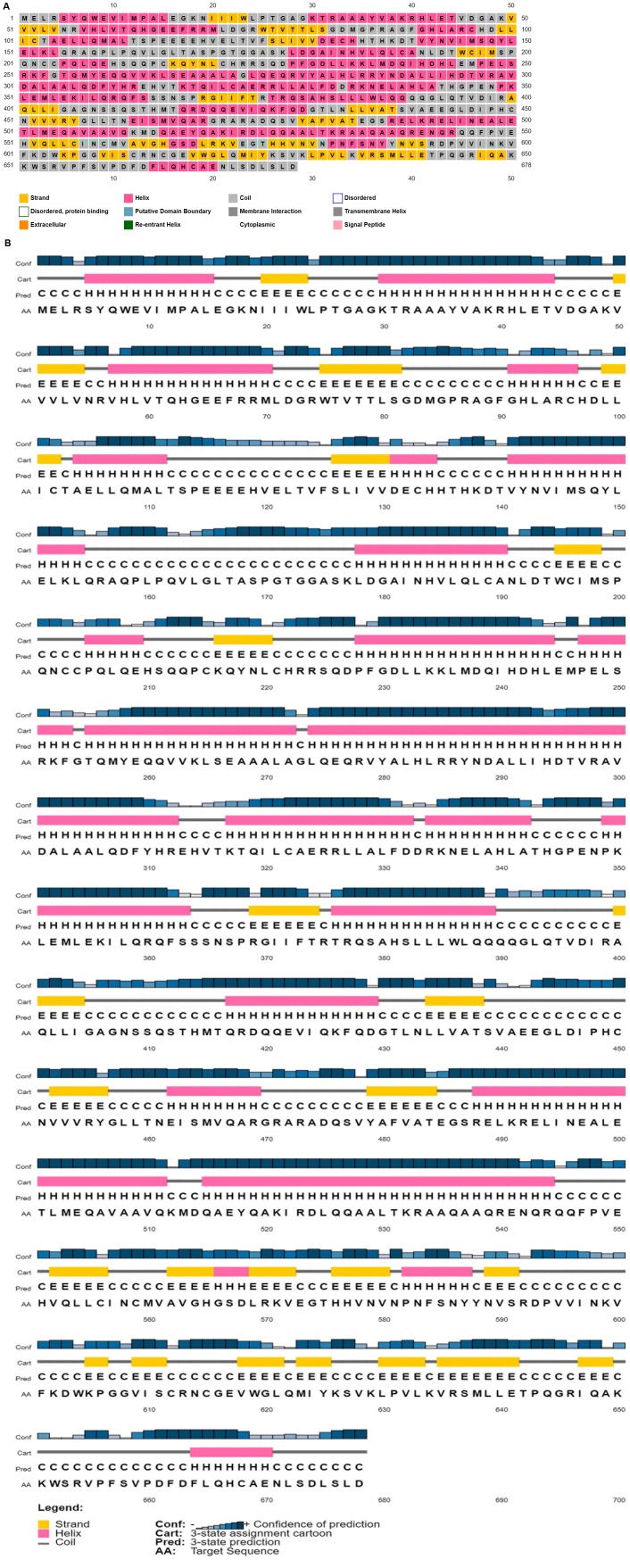
(A) PSIPRED result indicating the secondary structure and cellular location of the ATP-dependent RNA helicase; (B) Predicted three-state (helix/sheet/coil) secondary structure of ATP-dependent RNA helicase residues (AA) by PSIPRED at the confidence level (Conf); strands, helices, and coils are respectively represented as E, H, and C.

The secondary structure elements define a protein structure and their encoded fractions play a key role in designing various bioanalytical experiments. Using PSIPRED, the fraction of α-helix, coil and β-strands are orderly estimated to be 46.6, 37.4 and 15.9 (Figure 2A),and it indicates a substantial predominance of α-helix than the remaining elements. The estimated secondary structures are marked across the chain, along with their statistical confidence (Figure 2B).

### Functional analysis:

**Figure 3:**
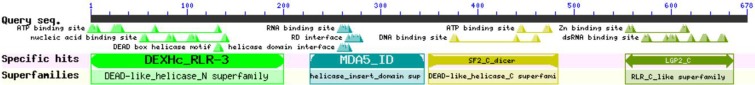
CDD results indicating sequence location and boundaries.

The sequence is functionally annotated through the InterPro server, and it is found to encode several signature motifs, viz. P-loop containing nucleoside triphosphate hydrolases (IPR027417), RIG-I like receptor, C-terminal domain superfamily (IPR038557), Helicase superfamily, 1/2 ATP binding domain (IPR014001), Helicase/UrvB, N-terminal (IPR006935), RIG-I receptor, C-terminal (IPR041204), Helicase C-terminal (IPR001650), RIG-I like receptor and C-terminal regulatory domain (IPR021673). The gene ontology search further confirms that the protein has DNA binding (GO: 0003677), ATP binding (GO: 0005524) and hydrolase activity (GO: 0016787). Moreover, the CDD database shows that the protein is encrypted with domains for four superfamilies viz. DEAD-like helicase_N_superfamily (cl28899), MDA5_ID (cd12090), SF2_C_dicer (cd18802) and LGP2_C (cd15806) (Figure 3).

**Figure 4:**
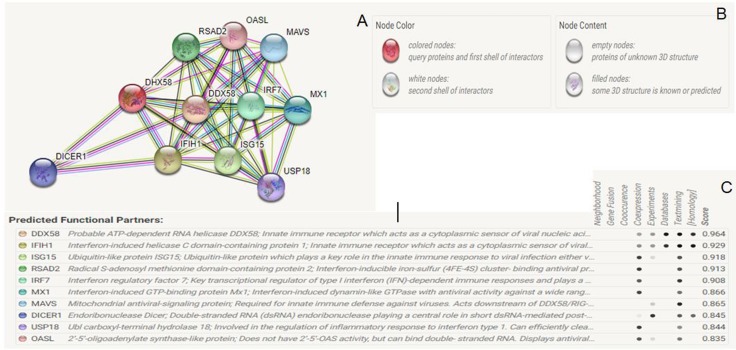
(a) The protein-protein network of the functionally interacting protein partners (b) Nodes and line color labels used to build the network (c) Estimated confidence scores of the STRING database for the interaction partners.

**Figure 5:**
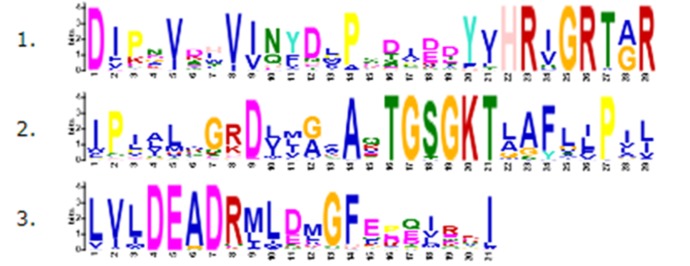
Conservation logo of the top-3 sequence motifs, extracted using the MEME suite, encoded in the ATP-dependent RNA helicase.

**Figure 6:**
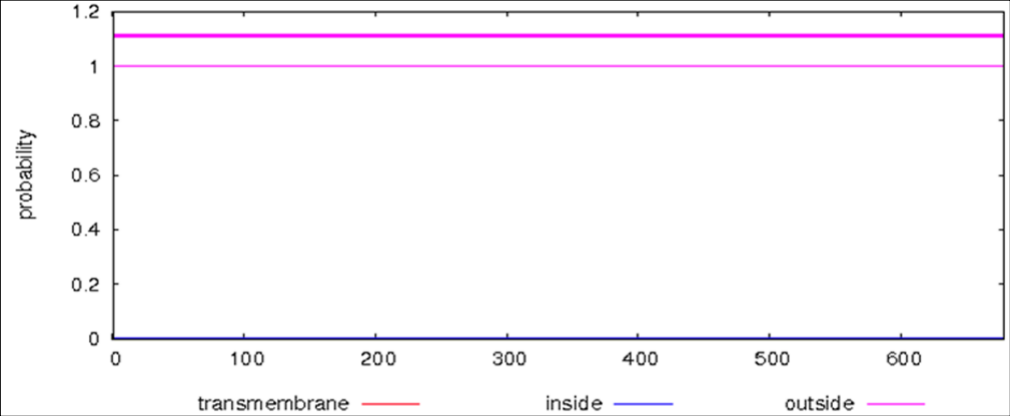
Graphical representation of the cellular location estimated by TMHMM for the ATP-dependent RNA helicase enzyme.

Through the STRING database, a resource of known and predicted protein-protein interactions, the top-ten potentially interacting partners are screened. The server ranks the functionally associated partners through an integrated confidence score by genome-wide network connectivity, and the ten partners show a score higher than 0.83. The protein DHX58 is identified to be an ATP-dependent RNA helicase. It lacks the cuspate activation and recruitment domain (CARD domain) and has its role in RIG-1 and MDA5-mediated signaling against the infectious virus or targeted cells. The predicted network of interacting protein partners shows a significantly higher confidence score of 0.964 for DDX58, an innate immune receptor. The network is constructed by retrieving data through the coexpression and published experimental results through textmining and extensive database screening. Further, the low-ranked partner 2'-5'-oligoadenylate synthetase like protein (OASL) shows a score of 0.835 (Figure 4). The proteins are known to actively participate in the immunological network of cellular proteins. The three most conserved motif sites, with the E-value scores of 1e-2683, 1.9e-2409 and5.9e-1728 are found for the ATP-dependent RNA helicase using the MEME server (Figure 5). The size of each logo character represents the evolutionary conservation of an amino acid at a specific site. The results reveal that the DEAD motif, associated with the ATP binding and hydrolysis, is encoded in the positions 4-7 in the third motif.TMHMM predicts the location of transmembrane, intracellular and extracellular regions, and it indicates that ATP-dependent RNA helicase is an extracellular protein (Figure 6). Further, to find the cleavage sites of extracellular digestive enzymes including caspase, trypsin, thermolysin, pepsin and proteinase K, the peptidecutter server is used. No cleavage sites are found for the caspase upstream and downstream enzyme, signifying the programmed cell death. However, 334 cleavage sites are found for proteinase K, an enzyme responsible for the degradation of nucleases.

### Molecular modeling:

HMM-profile is constructed through HHPred for the selected sequence, and the 5F9FE is found to be the top-ranked template structure. It shares a 53% similarity and completely spans the target sequence. On the basis of secondary structure features estimated by PSIPRED, the sequence alignment is manually curated and the selected sequence is modelled using MODELLER9.19, as per the strategies discussed earlier [46,47]. For resolving the non-physical atomic clashes, the predicted structure is iteratively refined through 3Drefine to extensively sample its conformational space. The refined structure orderly shows a credible TM-Score and Cɑ-RMSD of 0.96442 and 1.03 against 5F9FE. The model shows an ERRAT score of 94.1807, and it affirms the non-bonded interaction network in the model. The constructed decoy shows a DOPE and GA341 score of-77915.687 and 1.00 respectively. While the latter score indicates the structural compactness, the former energetic measure confirms the near-native credibility of the predicted model.

**Figure 7:**
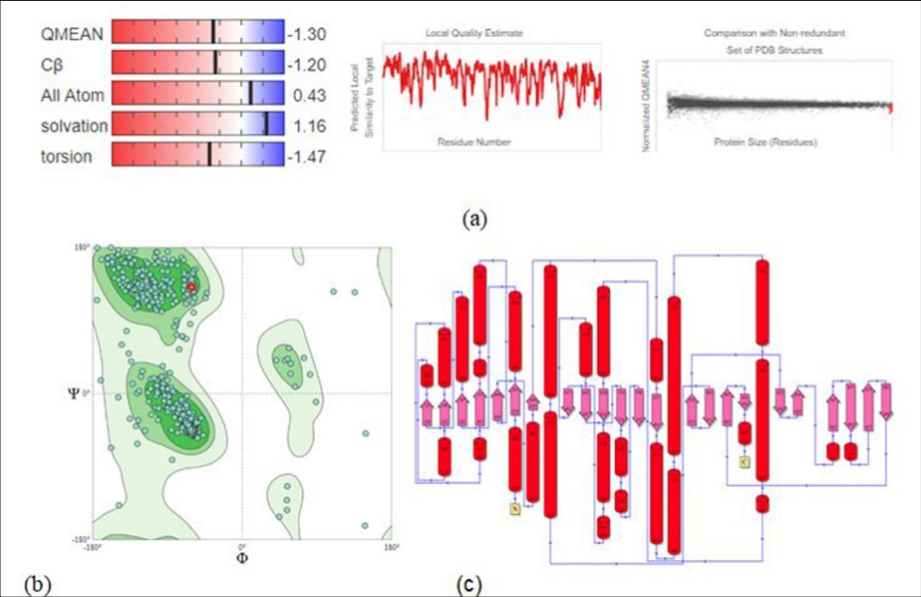
Structural assessment of the predicted protein model through (a) Qmean-score and Z score (b) PROCHECK-derived Ramachandran plot showing the 99.20% residues localized within the topologically allowed regions (c) Protein topology map, constructed using Profunc, with red color representing the helices and blue color representing the direction.

As shown in (Figure 7), a set of 90.70% and 7.50% residues are found to be localized within the most-favored and additionally allowed regions in the Ramachandran map, plotted through PROCHECK, as detailed in the following (Table 1) [48] Assessing through the QMEAN server, the model shows the clash score, rotamer outlier percentage and Molprobity score of 2.65, 0.52% and 1.48 respectively. It affirms the local and global accuracy and suggests that the topological accuracy of the predicted decoy is comparable to a medium-resolution crystallographic structure.

### Conservation and flexibility analysis:

**Figure 8:**
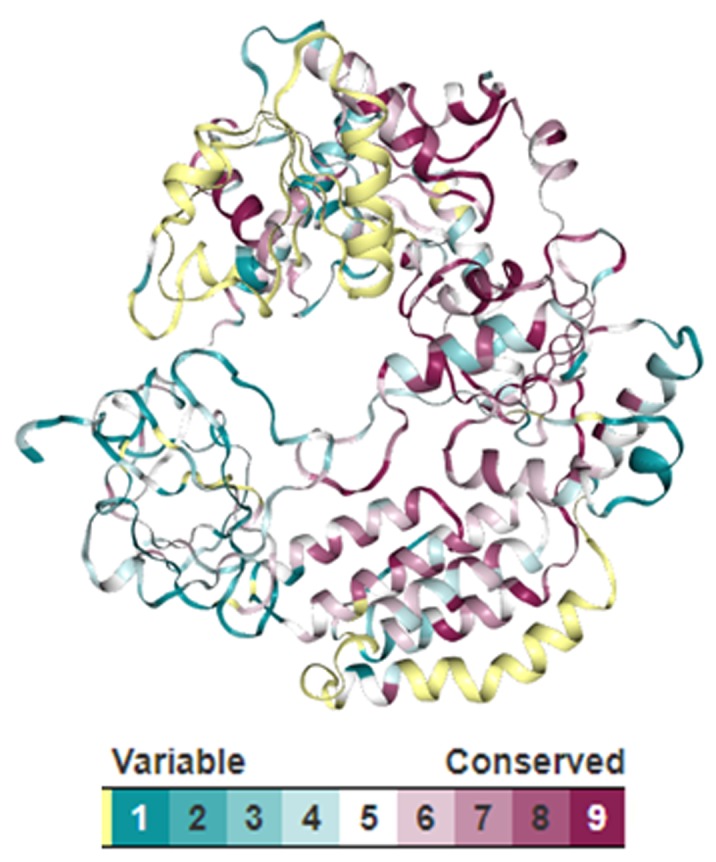
Consurf-derived conservation analysis of the human ATP-dependent RNA helicase. Color-coding is used to mark the evolutionary rate of residues over the predicted model. Low, mean and high evolutionary variability is orderly marked as maroon, white and turquoise.

The conservation level, indicating the color gradation with maroon, white and turquoise to orderly represent the higher, medium and lower order of sequence conservation, is mapped onto the surface of the constructed protein model (Figure 8). The analysis reveals an average pairwise distance score of 1.49264, within the range of 1.01758e^-07^ to 3.2305, across the entire sequence length. While only 68 residues are found to be completely conserved, 124, 308 and 178 loci are orderly found to be highly, moderately and poorly conserved.

**Figure 9:**
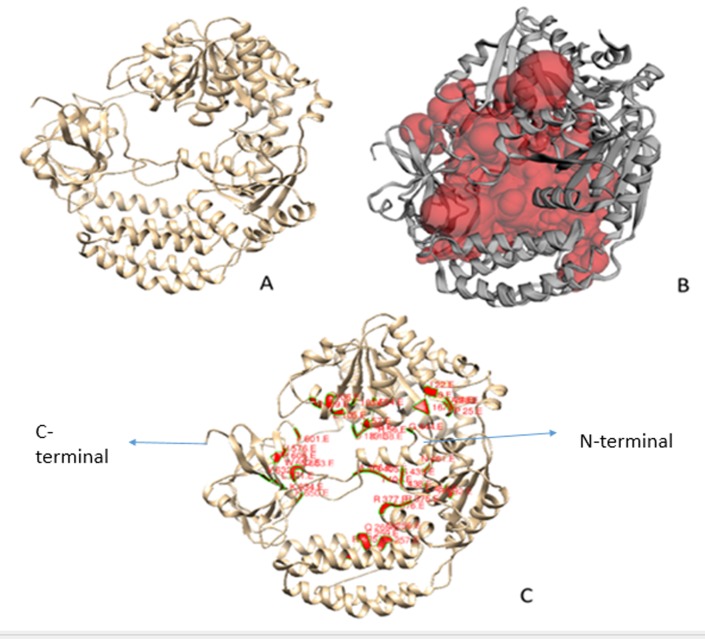
(A) Near-native model (B) Active-site zone estimated by CastP (C) Key active-site residues of the ATP-dependent RNA helicase (Q96C10.1).

The molecular surface of the helicase protein structure (Figure 9A) is analyzed through the CASTp server for identifying the pockets, cavities and cross channels. The biggest cavity shows the surface area and volume of 6077.513Å^2^ and 10513.441å^3^, and it signifies that the structure encodes a substantially broad cavity (Figure 9B). It is interesting to observe that only 42 residues (I22, L24, P25, A28, K30, V54, R56, V57, T103, E105, L106, M109, K138-D-T140, T167, Q256, M257, E259, Q260, R285, R375-T-R377, I404-G-A406, T438, S439, G444, L459, N461, R492, H576, F601, P606, L621, V632, K634, K650, W652, and S653) define the active site (Figure 9C). To estimate the atomic fluctuations across the cavity, the structural flexibility is estimated with the CABS-flex algorithm. On the basis of Calpha, Cβ, and side-chain representation, it quickly simulates a protein structure and overcomes the size limitation of the classical molecular dynamics strategy.

**Figure 10:**
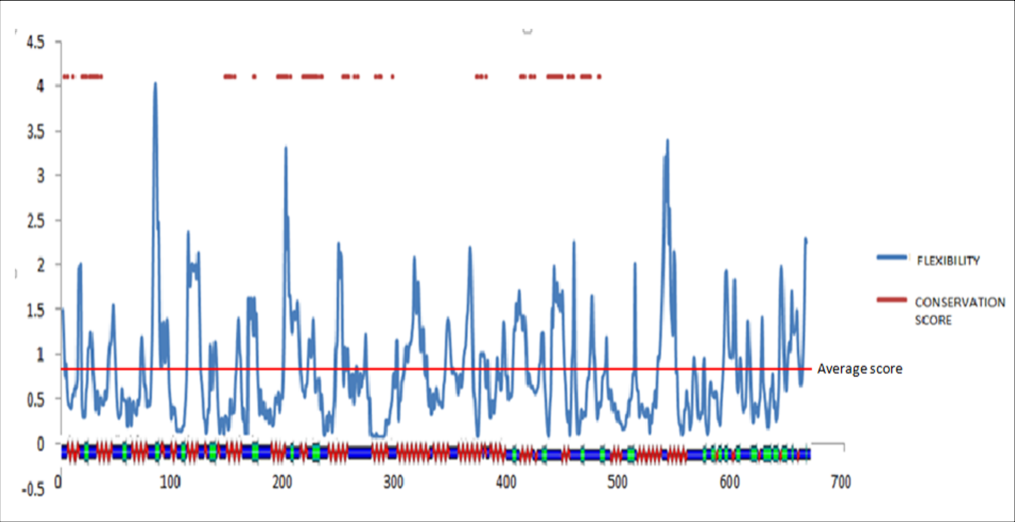
CABS-flex-estimated RMSF scores defined in correlation with their secondary structure and the sequence conservation (marked with red superficial bars) for the ATP-dependent RNA helicase. Several flexible residues are found to be evolutionarily conserved across the topologically important secondary structure elements.

For an input protein structure, the output ensembles the atomic-resolution profile representing the flexibility of the input structure. As the functionality of a protein is dependent on its topological flexibility, it is mandatory to map such vital sites across the protein sequence. Overlapping the sequence conservation map of this protein with the active-site cavity, it further illustrates that the core cavity is not highly conserved. Structural mapping through POLY VIEW-2D further shows that some flexible loci are significantly conserved and it delineates that these residues are essential for protein function. To analyze the residue flexibility score in correlation with the secondary structure and sequence conservation of the residues, the results are overlapped and the average structural fluctuations are marked with a red line (Figure 10). The average and standard deviation of the RMSF scores for these loci are orderly found to be 0.781 and 0.495, unlike the respective scores of 0.804 and 0.593 for the complete structure, and it indicates that the active site is a bit more structurally stabilized. However, only 9 residues (L24, A28, K30, Q256, R285, T438, S439, G444, and L459) are found to be conserved, and it shows that the flexibility is natively vital for only a few residues.

### Phylogeny analysis:

**Figure 11:**
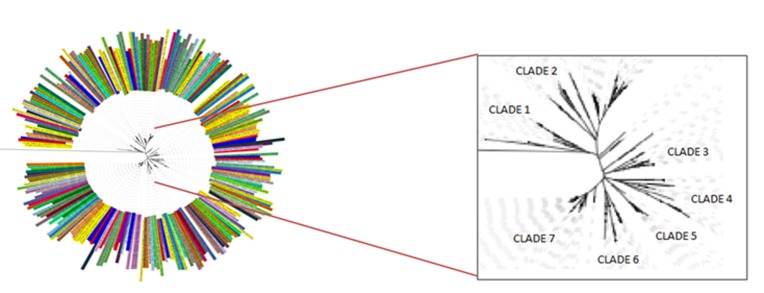
Phylogenetic tree of 259 sequences comprising 7 clades in which species in a clade are closely related to each other when compared to species in another clade.

The 259-sequence dataset is aligned through ClustalW and is curated by eliminating the poorly aligned positions and divergent regions. Gblocks server is used to select the informative positions of the sequences. The dataset shows a mutual sequence identity within the range of 13.32-99.87, and the lower limit indicates a distant evolutionary linkage. Excavating it further, it shows 7 major evolutionary clades, and orderly defines the subsets of 25, 70, 23, 27, 17, 26 and 62 sequences. The sequence-identity range for these clades lies within the range of 22.55-96.27, 27.1-99.87, 29.31-99.84, 23.49-99.84, 36.19-98.24, 33.33-95.03 and 32.36-99.83 respectively. The mean sequence identity for each of these clades is orderly found to be 40.37±16.62, 40.44±12.53, 45.77±16.29, 39.07±16.07, 58.50±16.33, 45.75±10.88 and 54.78±12.38. It thus indicates that the clade5 members are evolutionary too close and 27 clade4 members share a distant relationship. However, the species are uniformly present across most of the clades (Figure 11).

## Discussion:

The RNA helicase subfamily harbors several multi-functional enzymes. For its key role in various aspects of RNA metabolism, the ATP-dependent RNA helicase has been extensively studied [49]. However, the structural conservation and evolutionary divergence of several key sub familial members are still not functionally excavated through a functionally similar dataset [50]. While the sequence analysis of DHX58 protein reveals many characteristic features [51, 52],the STRING database has revealed DDX58 as the top-ranked partner, as also shown in the recently published reports [53]. DHX58 is further found to interact with IFIH1, ISG15, RSAD2, IRF7, MX1, MAVS, DICER1, USP18 and OASL. However, IFIH1 has been recently shown to have an affinity for RNA [54], and the helicase motif of DICER (DICER1) has been shown crucial for processing the siRNA [55]. Further, as shown by recent microarray analysis, 1.9 fold upregulation of DHX58 is orderly found associated with a 2.1, 2.2, 2.2, 2.4, 2.8 and 4.0 fold upregulation of MX1, IFIH1, USP18, DDX58, OASL, and RSAD2 proteins [56, 57]. Hence, in accordance with the earlier studies, our estimated network of the top-ranked proteins (Figure 4) strongly indicates a potential role of the interacting partners in the immune signaling mechanism of DHX58.Motif segments have only been shown to be highly conserved in contrast to a significant variability across the N- and C-terminal domains, majorly responsible to interact with a diverse set of proteins. However, our Consurf analysis shows statistically higher conservation for 168 residues through the HMM-profile of the constructed 259-sequence dataset, as mapped on the predicted near-native structure of DHX58 (Figure 8). Further, as estimated through CABS-flex (Figure 10), the structural fluctuations are found highest for some terminal residues, although the model shows a significant structural fluctuation across the chain. Although this is in agreement with the earlier results [58,59], it shows that the fluctuation of the key residues could possibly have a vital functional role.The evolutionary study shows a 7-clade evolutionary distribution of the constructed 259-sequence dataset, and each clade is found to span the sequences from all the available species. However, the structural superimposition based study of DEAD domains of DDX2A, DDX2B, DDX5, DDX10, DDX18, DDX20, DDX47, DDX52, and DDX53, and the helicase domains of DDX25 and DDX41 shows a Cα-RMSD within 0.6-1.9 Å over the diverse sequence identity range of 27%-86 [60]. The study thus adds on to the details reported earlier and it implicates that these protein structures are robustly conserved over the sequence alterations. Besides the interaction with a few molecules like β-catenin, a protein involved in the gene transcription [61], the active-sites of helicases have not been extensively excavated [62]. To extend it further, it is observed that the enzyme encodes a set of 42 active-site residues, of which 9 residues are found to be conserved. The active-site shows a lower topological fluctuation than the overall structure. Thus, the presented analysis provides a reliable framework for a more detailed evolutionary and structural analysis of ATP-dependent RNA helicase.

## Conclusions:

It is of interest to annotate the human protein Q96C10.1 using known data to model structure and infer function with potential role in the pathway. We document that ten proteins, including DDX58 and OASL are potential interacting protein partners with Q96C10.1. A dataset of 259 functionally similar homologs shows an evolutionary clustering within seven clades and shows conservation of only 9 active-site residues. It is inferred that active site residues are not highly conserved to link with the corresponding low structural similarity in these enzyme proteins.

## Figures and Tables

**Table 1:** PROCHECK estimated Ramachandran plot for the predicted helicase structure. It shows 99.2% residues in the favorable regions and confirms the modelling credibility.

Plot statistics	Number	Percentage
Residues in most favored regions	547	90.70%
Residues in additional allowed regions	45	7.50%
Residues in generously allowed regions	6	1.00%
Residues in disallowed regions	5	0.80%
Number of non-glycine and non-proline residues	603	100%
Number of end-residues (excl. Gly and Pro)	3	
Number of glycine residues (shown as triangles)	36	
Number of proline residues	24	
Total number of residues	666
